# Parotid lymphoepithelial cysts revealing HIV infection in a 12-year-old girl: A case report

**DOI:** 10.1016/j.amsu.2021.102338

**Published:** 2021-04-16

**Authors:** Salissou Iro, Ezzahra Hmoura, Faiçal Slimani

**Affiliations:** a Faculty of Medicine and Pharmacy, Hassan II University of Casablanca, Casablanca, Morocco; b Oral and Maxillofacial Surgery Department, CHU Ibn Rochd, Casablanca, Morocco

**Keywords:** Benign lymphoepithelial cysts, HIV, Diagnosis, Treatment

## Abstract

**Introduction:**

and importance: benign lymphoepithelial cysts are benign lesions consisting of one or more cysts of the salivary glands or neck regions that occur in 3–6% of patients with positive HIV serology. The objective of this work is to discuss the link between HIV and benign lymphoepithelial cysts.

**Case presentation:**

The authors report a case of benign lymphoepithelial parotid cysts in a 12-year-old girl who underwent a partial parotidectomy. HIV serology was performed in the patient and all her family and was positive only in the girl.

**Clinical discussion:**

Benign lymphoepithelial lesions of the parotid gland represent the main parotid pathology associated with HIV infection. They may be the first clinical manifestation of the virus. Diagnosis is often difficult, in most cases after surgical excision and histopathological evaluation of the mass. Treatment options include observation, highly active antiretroviral therapy (HAART), aspiration, sclerotherapy, and surgery.

**Conclusion:**

Because of the close relationship between parotid lymphoepithelial cysts and HIV infection, it is important to perform HIV serology in any patient with these types of cysts.

## Highlights

• Benign lymphoepithelial cysts are benign lesions consisting of one or more cysts of the salivary glands or neck regions.
• It occurs in 3-6% of patients with positive HIV serology.
• The presence of multiple intraparotid cysts should make one think of benign lymphoepithelial cysts.
• It is important to perform HIV serology screening in cases of BLEC in order to take appropriate surgical precautions.

## 1 Introduction

This work has been reported in line with the SCARE 2020 criteria [1].

Benign lymphoepithelial cysts (BLEC) are defined as rare and benign lesions consisting of one or more cysts of the salivary glands or the neck regions. They were first described in 1958 by Bernier et al. Their parotid location occurs in 3–6% of patients with positive HIV status [2]. They may also occur in the floor of the mouth, tonsils, thyroid gland, intrathoracic region, and pancreas [3]. Diagnosis is often difficult in most cases and is made after surgical excision and histopathological examination of the mass [4]. HIV testing should be performed routinely to make a diagnosis and initiate early treatment. Treatment options for parotid gland BLEC include surveillance, highly active antiretroviral therapy (HAART), aspiration, sclerotherapy, and surgery. The authors report here a Case of benign lymphoepithelial cysts of both parotid glands in a 12-year-old girl that was the origin of the diagnosis of HIV infection. The objective of this paper is to discuss the link between HIV and benign lymphoepithelial cysts.

**Fig. 1:**
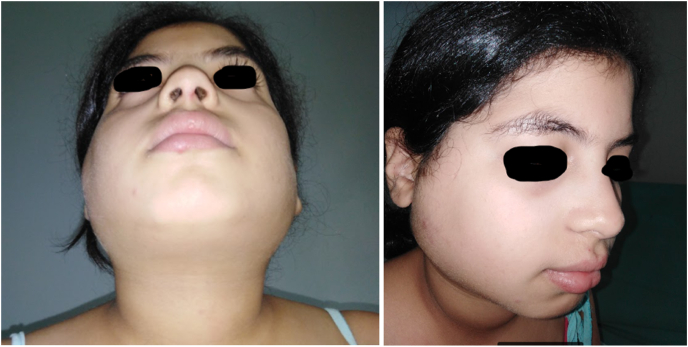
Pictures of the patient showing swelling of the 2 parotid regions.

**Fig. 2:**
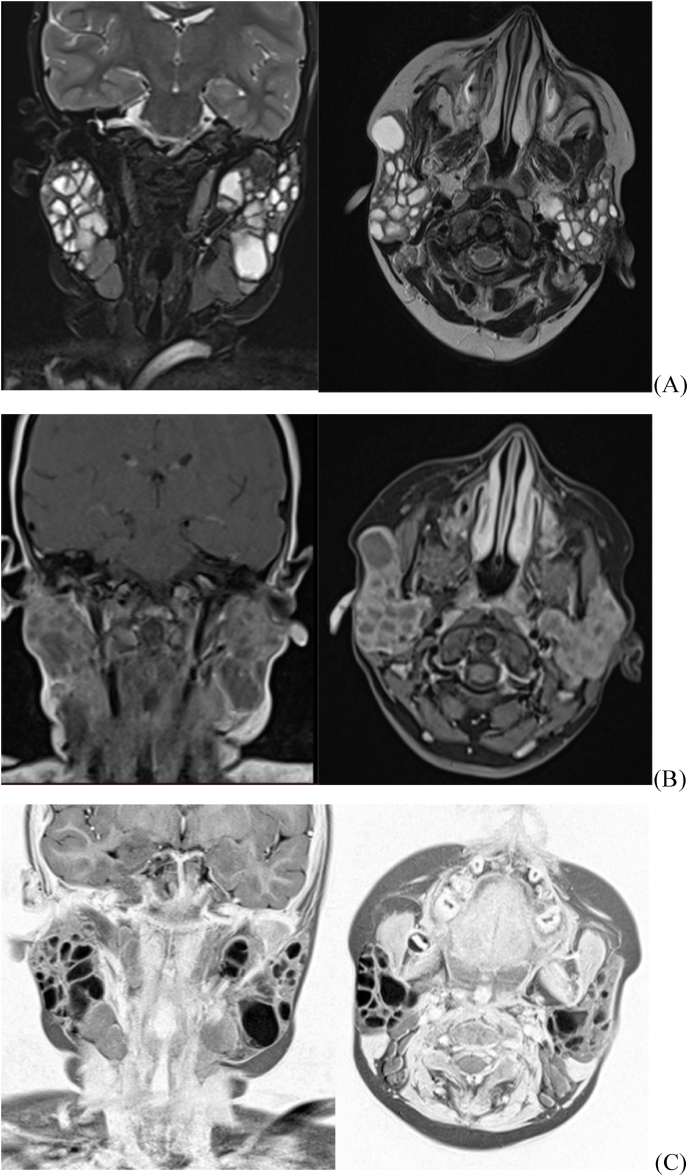
MRI pictures axial and coronal T2-weighted (A) T1-weighted (B) negative T2-weighted (C) showing enlarged parotid glands with multi-loculated cystic formations, the septa are of intermediate signal enhancing after gadulinium injection.

**Fig. 3:**
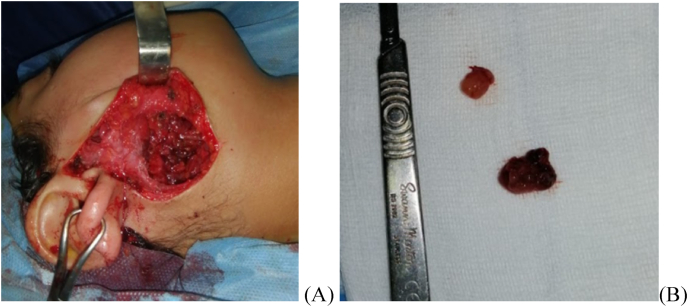
Intraoperative picture of the patient showing the approach (A) and the surgical specimen (B).

**Case presentation:** A 12-year-old girl was admitted with swelling of both parotid regions (Fig. 1). In the history of this patient, it was noted that she is the youngest of two children, from a non-consanguineous marriage, with a well-monitored pregnancy carried to term, medical delivery by the vaginal route, vaccinated according to the national immunization program, with good psychomotor development. Her family history includes a mother who died of a hepatic hydatid cyst. On examination, swellings in both parotid regions appeared since the age of 3 years, progressively increasing in size, without trismus, nor any notion of recurrent otitis. The clinical examination revealed a child in good general condition with a body mass index of 21.6 kg/m^2^. On examination of the right parotid region, a bilobed subcutaneous mass of soft consistency was noted, painless, mobile with the superficial and deep planes, about 5 cm in length, without any skin changes in view. In the left parotid region, the mass was moderately tender and of soft consistency, slightly mobile, and the skin opposite the mass was normal. The saliva was normal in appearance. Examination of the lymph nodes revealed multiple tender neck lymph nodes, mobile to the superficial and deep planes, the largest of which measured 1.5 cm in length. Several diagnoses were evoked in front of bilateral lesions of the parotid glands in a child without medical history. The radiological workup, including an ultrasound, showed bilateral parotiomegaly with multiple intraparotid cysts with fine echogenic content and vascularized septa, associated with bilateral subangulomandibular and jugulocarotid lymph nodes. Parotid Magnetic resonance imaging (MRI) showed enlarged parotid glands (Fig. 2), with lobular contours, the seat of multi-loculated cystic formations, involving superficial and deep lobes, with a pure liquid signal, whose septa are of an intermediates signal on T1 and T2 sequences, enhancing after gadolinium injection. A partial parotidectomy was performed under general anesthesia. Intraoperatively, cystic lesions (Fig. 3), measuring approximately 40 mm and 10 mm in diameter, were found. The cysts had no connection with the external auditory canal, without fistulas. The postoperative course was favorable, and the patient was put on preventive antibiotic therapy for 7 days, corticosteroid therapy at a dose of 40 mg per day for 5 days, and analgesics for 5 days. The patient was followed up at our routine specialized consultation 3, 6, and 12 months without any particularities. The histopathological analysis described salivary parenchyma, a cystic formation with a regular squamous lining without atypia, over a chorion rich in lymphocytes which are organized in lymphoid nodules, without tumor proliferation and concluded to benign lymphoepithelial cysts. HIV serology was performed on the child which was positive. The serology of the parents and the fry was negative. The child was referred to the pediatric infectious diseases department for further treatment, where she received a general evaluation of her disease and the initiation of a combination of antiretroviral therapy based on Efavirenz 600mg capsule, Lamivudine 150mg, Zidovudine 300mg, with periodic clinical and biological follow-up.

## 2 Discussion

Benign lymphoepithelial cysts are defined as rare and benign lesions consisting of one or more cysts of the salivary glands or neck regions. They were first described in 1958 by Bernier et al. Their parotid location occurs in 3–6% of patients with positive HIV status [2]. They may serve as the first clinical manifestation of the virus. They are more common in early HIV infection than in advanced AIDS [5]. Diagnosis often requires a history, physical examination, and aspiration biopsy for histopathological study [6].

Currently, there are two hypotheses about the pathophysiology of benign lymphoepithelial cysts. The first hypothesis is that they are multiple cysts within the lymph nodes trapped during embryogenesis of the parotid gland. HIV reactivated lymphatic proliferation occurs in the intraparotid lymph nodes. The glandular epithelium of the parotid gland becomes trapped in the normal intraparotid lymph nodes, resulting in cystic hypertrophy [7]. The second hypothesis is that HIV-infected cells migrate to the parotid glands. This migration triggers lymphoid proliferation and dysplasia of the salivary ducts, which induces an obstruction of the ducts; these cysts then form because of this obstruction [8].

Clinically, patients present with chronic swelling that is often bilateral, painless, and sometimes associated with xerostomia and polyadenopathy, indistinguishable from other parotid tumor pathologies [9]. These cysts do not invade surrounding structures and have no malignant potential, but may cause significant local disfigurement [10]. They rarely cause facial paralysis and xerostomia, and if the patient presents with systematic symptoms, such as fever, weight loss, or night sweats, one should look for tuberculosis or lymphoma, they can also be found in the floor of the mouth, tonsils, thyroid gland, intrathoracic region, and pancreas [3]. The diagnosis of BLEC is made based on medical history, physical examination, and biopsy. Non-invasive diagnostic modalities include ultrasound, CT scan, and MRI, which can detect multiple thin-walled cysts with diffuse neck lymph nodes. Invasive diagnostic modalities include a fine-needle biopsy or cytopuncture and parotid gland biopsy, which can rule out differential diagnosis [11]. **These differential diagnoses are: first branchial cleft cyst, Warthin's tumor, Goujerot-Sjogren's syndrome, sialocele, sarcoidosis, necrotic intraparotid lymph nodes, parotid enlargement. In children, lymphoma and metastases can be added.** In this patient, a partial parotidectomy for diagnostic purposes was opted for, and the anatomopathological study of the surgical specimen allowed the diagnosis of BLEC.

The origin of this HIV infection in this girl has remained mysterious. Since the serology of the parents and siblings is negative, rape or sexual abuse was thought to be the cause. But the interrogation by a child psychiatrist and the gynecological examination did not detect any clue.

Histopathologically, lymphoepithelial cysts usually consist of multiple benign epithelial cysts accompanied by dense lymphoid tissue. The epithelial lining may be squamous, cuboidal, or columnar. Although some cysts have a pseudostratified epithelium, the stratified squamous epithelium is the most common. Lymphoid tissue replaces normal parotid parenchyma, and the lymphoid follicles are larger and more irregularly shaped than the follicles of normal lymph nodes [12]. The germinal centers often contain numerous macrophages, and some germinal centers contain small attenuated lymphocytes that invaginate into the follicles [13].

Treatment of lymphoepithelial cysts has been debated and varies between isolated and HIV-associated lymphoepithelial cysts [3]. The finding of BLEC of the parotid gland is not in itself an indication for antiretroviral combination therapy. Antiretroviral combination therapy should be initiated in the presence of a CD4 T-cell count of fewer than 500 mm^3^ in the absence of opportunistic infections [14]. HIV-positive patients with BLEC have several options, such as repeated aspiration and drainage, radiation therapy, sclerotherapy, highly active antiretroviral therapy (HAART) drug therapy, and surgery.

Repeated aspiration and drainage of cysts have been shown to have a 100% recurrence rate in weeks to months [15]. Sclerotherapy is an option where cysts are injected with sclerosing agents, such as sodium morrhuate, doxycycline, ethanol, bleomycin, and picibanil [11].

Highly active antiretroviral therapy (HAART) is an effective option for treating HIV-associated lymphoepithelial cysts, with studies showing a decrease in the size or elimination of parotid cysts after its initiation [8], the authors suggest, in HIV-positive patients, to start treatment with highly active antiretroviral therapy (HAART) before considering a more aggressive approach [16]. Initiation of antiretroviral therapy in children is similar to that of adults; in fact, all HIV-infected children should receive antiretroviral therapy as soon as possible (rapid onset, within 1–2 weeks of diagnosis) [17]. Yet, some patients choose surgery because recurrence of BLEC is possible with other treatment options. Some authors consider parotidectomy to be the gold standard treatment because it is the only method that provides a complete response without recurrence [8]. However, some do not recommend surgery as first-line treatment, due to the bilateral and progressive nature of the disease, the 2–7% risk of facial nerve damage, bleeding [18]. Therefore, surgery may be a good option in cases of malignancy or poor response to other treatment options.

In the Case reported in this work, the child, after evaluation of her disease, was put on antiretroviral combination therapy, with periodic monitoring every 3 months.

There is no consensus on the modality of monitoring, but clinical and biological follow-up is important to detect treatment toxicity and failure and to assess compliance. Benign lymphoepithelial lesions in association with HIV sometimes evolve into B-cell lymphoma, which should be evoked in Case of growth of a lesion, contemporaneous with asthenia, sweating, or fever [14].

## 3 Conclusion

Given the close relationship between the presence of lymphoepithelial cysts and HIV infection, it is important to perform screening HIV serology once the diagnosis is made. The presence of multiple intraparotid cysts should make one think of BLEC and therefore HIV serology to take adequate surgical precautions. Treatment is usually conservative, with surgery indicated if there is a poor response to other treatment options.

## Conflicts of interest

Authors of this article have no conflict or competing interests. All of the authors approved the final version of the manuscript.

## Sources of funding

The authors declared that this study has received no financial support.

## Ethical approval

Written informed consent was obtained from the patient's parent for publication of this Case report and accompanying images. A copy of the written consent is available for review by the Editor-in-Chief of this journal on request.

## Consent

Written informed consent was obtained from the patient for publication of this Case report and accompanying images. A copy of the written consent is available for review by the Editor-in-Chief of this journal on request.

## Author contribution

Salissou Iro: Corresponding author writing the paper.

Ezzahra Hmoura: writing the paper.

Faiçal Slimani: Correction of the paper.

## Registration of research studies

1. Name of the registry: research registry.

2. Unique Identifying number or registration ID: 6658.

3. Hyperlink to your specific registration (must be publicly accessible and will be checked):

## Guarantor

IRO SALISSOU
